# A Tribute to Abe Mickal, 1912–2001

**DOI:** 10.1155/S1064744901000308

**Published:** 2001

**Authors:** Sebastian Faro


### A tribute to Abe Mickal, 1912–2001

**Sebastian Faro**

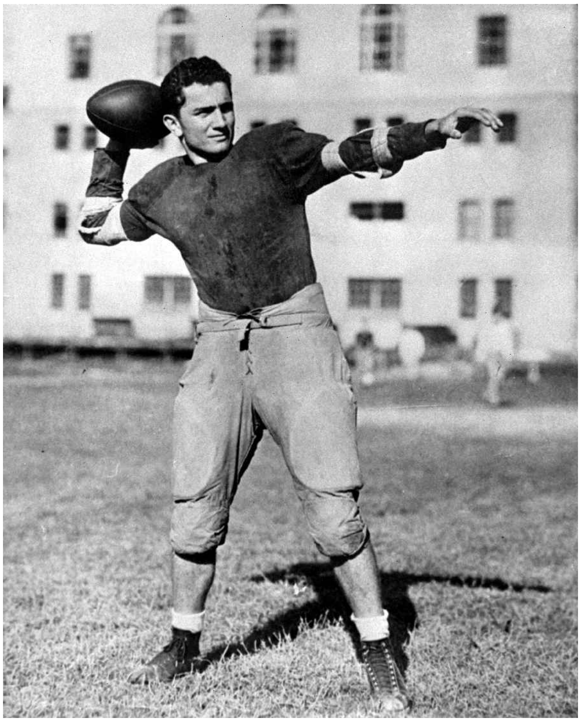

*Picture courtesy of I.S.U. Sports Information*

Abe Mickal was born in Talia, Lebanon in 1912 and moved to the United States in 1920 to live in McComb, Mississippi. Among his accomplishments, he was an outstanding high school and college football player at Louisiana State University. He was named all-southeastern conference and second-team all-American in 1934. In 1967 he was elected to the College Football Hall of Fame. He was also elected to the LSU Football Hall of Fame, Louisiana Sports Hall of Fame, Mississippi Sports Hall of Fame, Greater New Orleans Sports Hall of Fame, and the National Football Foundation Hall of Fame.

Dr. Mickal obtained his medical degree from LSU School of Medicine in 1940. He served as a major in the US Army during World War II. After military service, he completed his residency in Obstetrics and Gynecology at Charity Hospital from 1946–1949. He was Professor and Chairman of the Department of Obstetrics and Gynecology at Louisiana State University from 1959 until 1980, then became Chairman Emeritus.

Abe Mickal had a distinguished career in obstetrics and gynecology. He served as president of the New Orleans Gynecological and Obstetrical Society, the Association of Professors of Gynecology and Obstetrics and the Society of Gynecologic Surgeons, and was a founding member of the Society of Gynecologic Oncologists.

Dr. Mickal was a true teacher who trained numerous physicians, taking a sincere interest in the development and careers of each resident. There is no doubt he was an inspiration to many young physicians. Dr. Mickal had exceptional clinical skills and imparted this knowledge to his residents and faculty. An involved Chairman who put his students, residents, and faculty development before his own career, he was always available to assist physicians at any time. He could often be found on the wards at Charity Hospital with his medical students and residents sharing his knowledge and skills.

Abe Mickal was instrumental in fostering the development of infectious diseases in obstetrics and gynecology. He personally assisted in the development of clinical anaerobic microbiology in the Departments of Medicine and Obstetrics and Gynecology at LSU. He had a sincere and deep interest in infectious diseases, and was both a loyal supporter of the Infectious Disease Society for Obstetrics and Gynecology and assisted in its development.

Dr. Abe Mickal had a significant impact on the field of Obstetrics and Gynecology, and the subspeciality of infectious diseases. There is no doubt he will truly be missed.

